# Supplementary material for: Global Trends in Research on Cell-Free Nucleic Acids in Obstetrics and Gynecology during 2017–2021
Source: J Clin Med. 2022 Sep 22;11(19):5545. doi: 10.3390/jcm11195545 (PMC9572904; doi:10.3390/jcm11195545)
Supplement: Supplementary file 1 [file jcm-11-05545-s001.zip › Supplementarty Table S1.pdf]

Supplementary Table S1 High-frequency MeSH terms/MeSH subheadings from the included papers on cell-free nucleic acids

Rank.	Major MeSH terms/ MeSH subheadings	Cluster	Frequency	Proportion of frequency (%)	Cumulative percentage (%)
1	Cell-Free Nucleic Acids/blood	2	639	3.8538	3.8538
2	Circulating Tumor DNA/genetics	1	572	3.4497	7.3035
3	Circulating MicroRNA/blood	5	501	3.0215	10.3251
4	Circulating Tumor DNA/blood	0	448	2.7019	13.0270
5	Cell-Free Nucleic Acids/genetics	2	439	2.6476	15.6746
6	Carcinoma, Non-Small-Cell Lung/genetics	1	359	2.1651	17.8397
7	Circulating MicroRNA/genetics	5	257	1.5500	19.3897
8	Liquid Biopsy/methods	4	195	1.1760	20.5657
9	Cell-Free Nucleic Acids/analysis	2	190	1.1459	21.7116
10	Colorectal Neoplasms/genetics	0	157	0.9469	22.6585
11	Breast Neoplasms/genetics	5	152	0.9167	23.5752
12	Carcinoma, Non-Small-Cell Lung/drug therapy	1	131	0.7901	24.3652
13	High-Throughput Nucleotide Sequencing/methods	1	122	0.7358	25.1010
14	Circulating Tumor DNA/analysis	4	118	0.7117	25.8127
15	Prenatal Diagnosis/methods	2	117	0.7056	26.5183
16	Antineoplastic Combined Chemotherapy Protocols/therapeutic use	1	106	0.6393	27.1576
17	Cell-Free Nucleic Acids/metabolism	3	103	0.6212	27.7788
18	Real-Time Polymerase Chain Reaction/methods	1	102	0.6152	28.3939
19	Carcinoma, Non-Small-Cell Lung/diagnosis	4	96	0.5790	28.9729
20	Carcinoma, Non-Small-Cell Lung/blood	1	89	0.5368	29.5097
21	Carcinoma, Non-Small-Cell Lung/pathology	1	87	0.5247	30.0344

22	Circulating MicroRNA/metabolism	5	82	0.4945	30.5289
23	Carcinoma, Hepatocellular/genetics	4	81	0.4885	31.0174
24	Early Detection of Cancer/methods	4	77	0.4644	31.4818
25	Breast Neoplasms/diagnosis	5	74	0.4463	31.9281
26	Prostatic Neoplasms, Castration-Resistant/genetics	3	71	0.4282	32.3563
27	Proto-Oncogene Proteins p21(ras)/genetics	0	67	0.4041	32.7604
28	Colorectal Neoplasms/blood	0	65	0.3920	33.1524
29	Colorectal Neoplasms/diagnosis	0	63	0.3800	33.5324
30	Neoplastic Cells, Circulating/pathology	4	61	0.3679	33.9002
31	Genetic Testing/methods	2	57	0.3438	34.2440
32	Exosomes/metabolism	5	55	0.3317	34.5757
33	Pancreatic Neoplasms/genetics	0	53	0.3196	34.8954
34	Breast Neoplasms/blood	5	52	0.3136	35.2090
35	Noninvasive Prenatal Testing/methods	2	52	0.3136	35.5226
36	Down Syndrome/diagnosis	2	50	0.3015	35.8241
37	Cell-Free Nucleic Acids/isolation & purification	4	48	0.2895	36.1136
38	DNA Mutational Analysis/methods	1	46	0.2774	36.3910
39	Cell-Free Nucleic Acids/urine	2	45	0.2714	36.6624
40	Proto-Oncogene Proteins B-raf/genetics	0	45	0.2714	36.9338
41	Breast Neoplasms/drug therapy	5	44	0.2654	37.1992
42	Extracellular Vesicles/metabolism	5	44	0.2654	37.4646
43	Circulating MicroRNA/analysis	5	44	0.2654	37.7299
44	Drug Resistance, Neoplasm/genetics	1	44	0.2654	37.9953
45	Breast Neoplasms/pathology	5	44	0.2654	38.2607

46	Trisomy 13 Syndrome/diagnosis	2	41	0.2473	38.5079
47	Sequence Analysis, DNA/methods	1	40	0.2412	38.7492
48	Stomach Neoplasms/genetics	3	38	0.2292	38.9783
49	Graft Rejection/diagnosis	2	38	0.2292	39.2075
50	Exosomes/genetics	5	37	0.2231	39.4307
51	Pancreatic Neoplasms/diagnosis	0	37	0.2231	39.6538
52	Colorectal Neoplasms/drug therapy	0	36	0.2171	39.8709
53	DNA, Mitochondrial/blood	2	35	0.2111	40.0820
54	Chromosome Disorders/diagnosis	2	35	0.2111	40.2931
55	Carcinoma, Hepatocellular/diagnosis	4	35	0.2111	40.5042
56	Colorectal Neoplasms/pathology	0	34	0.2051	40.7092
57	ErbB Receptors/genetics	1	34	0.2051	40.9143
58	RNA, Long Noncoding/genetics	4	33	0.1990	41.1133
59	Genomics/methods	4	32	0.1930	41.3063
60	Kidney Transplantation/adverse effects	2	32	0.1930	41.4993
61	Gene Expression Profiling/methods	5	31	0.1870	41.6863
62	Circulating Tumor DNA/isolation & purification	1	31	0.1870	41.8732
63	Neoplasm Recurrence, Local/diagnosis	3	31	0.1870	42.0602
64	Prostatic Neoplasms/diagnosis	3	30	0.1809	42.2411
65	Circulating Tumor DNA/metabolism	0	30	0.1809	42.4220
66	Fetus/metabolism	2	30	0.1809	42.6030
67	Melanoma/genetics	3	30	0.1809	42.7839
68	Protein Kinase Inhibitors/therapeutic use	1	28	0.1689	42.9528
69	DNA Methylation/genetics	4	28	0.1689	43.1216

70	RNA, Long Noncoding/blood	5	27	0.1628	43.2845
71	Circulating Tumor DNA/cerebrospinal fluid	4	25	0.1508	43.4353
72	Prostatic Neoplasms/blood	3	25	0.1508	43.5860
73	Precision Medicine/methods	4	24	0.1447	43.7308
74	Neoplasm Recurrence, Local/blood	0	23	0.1387	43.8695
75	Ovarian Neoplasms/diagnosis	3	23	0.1387	44.0082
76	Liquid Biopsy/standards	4	22	0.1327	44.1409
77	Urinary Bladder Neoplasms/genetics	2	22	0.1327	44.2736
78	Adenocarcinoma of Lung/genetics	1	22	0.1327	44.4062
79	Stomach Neoplasms/diagnosis	3	22	0.1327	44.5389
80	Extracellular Traps/metabolism	3	21	0.1267	44.6656
81	Cell-Free Nucleic Acids/chemistry	3	21	0.1267	44.7922
82	Urinary Bladder Neoplasms/diagnosis	2	21	0.1267	44.9189
83	Brain Neoplasms/diagnosis	4	20	0.1206	45.0395
84	Coronary Artery Disease/blood	5	20	0.1206	45.1601
85	Carcinoma, Pancreatic Ductal/genetics	0	20	0.1206	45.2807
86	Pre-Eclampsia/diagnosis	2	20	0.1206	45.4014
87	Diabetes Mellitus, Type 2/blood	5	20	0.1206	45.5220
88	5-Methylcytosine/analogs & derivatives	3	19	0.1146	45.6366
89	Whole Genome Sequencing/methods	2	19	0.1146	45.7512
90	Prostatic Neoplasms/pathology	3	19	0.1146	45.8657
91	Class I Phosphatidylinositol 3-Kinases/genetics	5	19	0.1146	45.9803
92	Biosensing Techniques/methods	0	19	0.1146	46.0949
93	Esophageal Neoplasms/genetics	3	18	0.1086	46.2035

94	Melanoma/blood	0	18	0.1086	46.3120
95	Melanoma/diagnosis	3	18	0.1086	46.4206
