# Supplementary material for: Global Trends in Research on Cell-Free Nucleic Acids in Obstetrics and Gynecology during 2017–2021
Source: J Clin Med. 2022 Sep 22;11(19):5545. doi: 10.3390/jcm11195545 (PMC9572904; doi:10.3390/jcm11195545)
Supplement: Supplementary file 1 [file jcm-11-05545-s001.zip › Supplementarty Table S2.pdf]

Supplementary Table S2 Cluster analysis of high-frequency major MeSH terms/MeSH subheadings of cell-free nucleic acids

Cluster	Number of MeSH terms*	Cluster analysis
0	29,10,52,65,92,40,27,85,33,51,56,28,4,74,94	1. Colorectal and pancreatic neoplasms genetics: Proto-Oncogene Proteins p21(ras) and B-raf genetics<br>2. Early detection of Cancer: biosensing techniques
1	44,12,68,16,21,57,6,78,20,62,18,47,13,2,38	1. DNA methylation and mutational analysis methods: high throughput nucleotide sequencing<br>2. Lung neoplasms drug therapy and antineoplastic agents: protein kinase inhibitors
2	82,77,39,60,49,1,53,86,5,89,35,46,36,15,54,31,9,66	1. Cell-free nucleic acids sequence analysis for noninvasive prenatal testing<br>2. Cell-free nucleic acids for genetic testing: chromosome disorders, prenatal, pre-eclampsia, Down syndrome diagnosis, Trisomy 13 syndrome and fetus metabolism tests<br>3. Cell-free nucleic acids isolation and purification<br>4. Urinary Bladder Neoplasms genetics and diagnosis
3	67,95,64,26,90,72,75,63,93,48,79,81,88,17,80	1. Melanoma, prostatic, esophageal and stomach neoplasms genetics and diagnosis<br>2. Cell-free nucleic acids metabolism
4	73,59,37,24,14,30,76,8,83,71,58,69,23,55,19	1. Precision Medicine<br>2. Circulating tumor RNA metabolism and analysis<br>3. Hepatocellular and non-small-cell lung carcinoma genetics and diagnosis
5	43,50,7,61,87,84,3,32,22,42,70,34,45,91,25,11,41	1. Circulating neoplastic cells metabolism<br>2. Breast neoplasms genetics and diagnosis<br>3. Exosomes, circulating microRNA and lcnRNA genetics

4. Coronary artery disease, type 2 diabetes mellitus diagnosis

*Represents the serial number of high-frequency major MeSH terms/MeSH subheadings shown in Supplementary Table 1.
